# Health profile of people living in the Gare Palma mining area of Tamnar block, Raigarh, Chhattisgarh, India

**DOI:** 10.3389/fpubh.2023.1010025

**Published:** 2023-03-21

**Authors:** Suyesh Shrivastava, Ravindra Kumar, Sirin Khan, Arvind Kavishwar, Ashok Gupta, Harpreet Kaur, Madhuchanda Das, Ravendra Sharma, Tapas Chakma

**Affiliations:** ^1^ICMR-National Institute of Research in Tribal Health, Jabalpur, India; ^2^Indian Council of Medical Research, New Delhi, India; ^3^Indian Council of Medical Research National Institute of Medical Statistics (NIMS), New Delhi, India

**Keywords:** morbidity, risk factors, tribals, Chhattisgarh, mines (industrial sites)

## Abstract

**Introduction:**

A community-based health survey was conducted in Tamnar block, Raigarh district of Chhattisgarh, India.

**Methodology:**

A total of 909 individuals (adults) were selected from 909 households from 33 sampled villages from March 2019 to February 2020. All individuals were clinically examined, and observations were recorded.

**Results:**

Among adults older than 18 years, hypertension was observed in 21.7%. Type II diabetes was observed in only 4.0% of individuals. Tuberculosis was seen in 23 (2.5%) individuals.

**Discussion:**

Common morbidities were similar in tribal and non-tribal communities living in the same area. For communicable diseases, being male, having nutritional deficiencies, and smoking were independent risk factors. For non-communicable diseases, the independent significant risk factors identified were being male, an altered body mass index, disturbed sleep, smoking, and nutritional deficiencies.

## Introduction

Chhattisgarh is home to 7.5% of the tribes of India, and tribal communities comprise 33.84% of the Raigarh district of Chhattisgarh. The main tribes inhabiting Raigarh district are Birhor, Kawar, and Binjwar (1), who speak the local dialects of Chhattisgarh. Raigarh district, especially the Gare Palma area, is abundant in important minerals like coal, quartzite, limestone, and dolomite. Tamnar (22.08°N, 83.44°E) and an adjacent block Gharghoda have 13 coal mines and 12 power plants, which continuously emit fly ash, leading to poor air quality. The majority of the population of these blocks work in these mines. Apart from pollution from the power plants, residents of Tamnar block are also exposed to particulate matter by more than 5,000 heavy coal-carrying vehicles every day around the clock. These coal-carrying vehicles not only cause air pollution but also create noise pollution, adversely affecting the health of nearby residents.

Mining activities put the residents at increased risk of diseases such as acute respiratory infection (ARI), tuberculosis, gastro-intestinal diseases, skin disease, and trauma (2). Apart from environmental and occupational health hazards, undernutrition and changing lifestyles make residents vulnerable to various other diseases (2). Furthermore, the status of healthcare resources in Tamnar block is poor. There is only one community health center (CHC) and one primary healthcare center (PHC), and these health centers are operating without specialist doctors. As well as the shortage of doctors, 46.6% of nurses and 30% of paramedical posts are vacant at these facilities, thereby worsening the situation further. Therefore, most of the residents in these areas cannot easily avail of the basic primary healthcare services from the centers.

The knowledge of predominant morbidities in an industrial area is crucial for the formulation of diagnostic, preventive, and management strategies by policymakers. We undertook the study at the request of the Ministry of Environment and Forests, Government of India, to evaluate the health status of individuals living around these mining areas.

## Methods

### Study design

This cross-sectional study was conducted among adults (age > 15 years) from March 2019 to February 2020 to evaluate the disease profile of residents of Tamnar block of Raigarh district, Chhattisgarh, India.

### Study setup

This is a community-based study.

### Sample size

In the census of 2011, the total population of Tamnar block was 97,975. In 2018, we assumed the population to have increased by 10%, leading to an approximate population of 107,772. Since there was no reference literature available, we assumed that at least 50% of individuals suffered from some type of morbidity. Using the Right Size statistical software of CDC, Atlanta, a cluster size of 20, a 90% confidence interval, and a 0.3 rate of homogeneity with a design effect of 6.7, a total of about 660 responses were required from 33 clusters. Assuming 25% non-responders, a minimum of 825 individuals were required for this cross-sectional study.

### Sampling design

**Figure 1:**
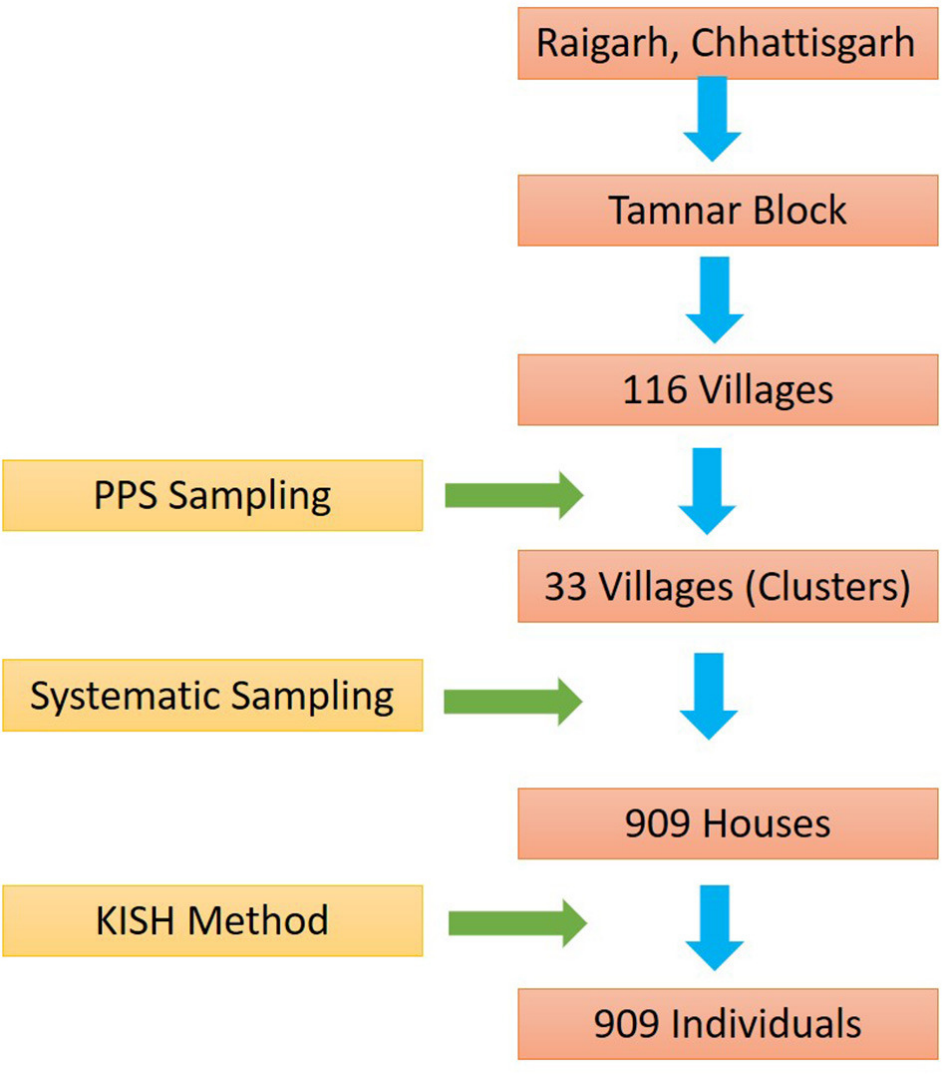
Overview of sampling design. H.H viz Household.

Of 116 villages of Tamnar block, 33 villages were selected based on population proportionate to size (PPS). The systematic approach to house selection (Kth house depending upon the size of the village) was adopted. At least 20 houses were selected from each village. A total of 909 houses were selected for the study. After selecting the house, an adult individual from each house was selected. Men were also interviewed for the study but the maximum participants were women as they were available at the time of survey. The complete sampling methodology adopted is shown in Figure 1.

### Training

All staff of the project, including the medical officer, field investigators, and laboratory technicians, were trained by the Principal Investigator (PI) and Co- Principal Investigator (Co-PI) of the project and standardized for clinical and laboratory investigations at Indian Council of Medical Research National Institute of Research in Tribal Health (ICMR-NIRTH) Jabalpur for a period of 2 weeks.

### Data collection

Demographic and socioeconomic particulars such as age, gender, ethnicity, occupation, income, type of house, alcohol, and tobacco intake were recorded from all 909 individuals by personal interview in a pretested and pre-coded pro forma.

### Clinical examination

After obtaining written informed consent, 909 individuals were clinically examined (within 15 days preceding data collection) by a medical officer for the presence of current morbidities such as fever, diarrhea, acute respiratory infection, and anemia, which were recorded. Nutritional deficiency disorders were also recorded. A history of chronic diseases and conditions, such as hypertension, diabetes, tuberculosis, sickle cell disease, cerebro-cardiovascular events, and chronic kidney disease, was recorded based on personal interviews and/or previous medical records obtained from an authorized medical practitioner. Operational definitions used in the study are described in detail in the Supplementary material.

### Anthropometric measurements

Height and weight were measured following standard procedures using an anthropometric rod (SECA, Germany) and digital weighing machine (SECA, Germany), respectively. The study participants were categorized into different body mass index (BMI) groups as per Asian Indian Guidelines (Supplementary material) (3). Emaciation is considered the most severe variety of malnutrition. In our study, an individual is considered emaciated when their BMI is ≤ 18.5.

### Laboratory investigations

A spot malaria test was done using a commercially available rapid diagnostic kit (Standard Diagnostics, Inc., Republic of Korea) and by microscopy on participants who had a fever on the day of examination. Random peripheral capillary blood sugar was measured by using a commercially available blood glucometer (CodeFree, SD Biosensor, Republic of Korea) with participants having signs and symptoms of type II diabetes. Spot and morning sputum samples were collected from symptomatic individuals. Smear was made in the field, and acid-fast bacilli (AFB) examination was done. X-rays of patients with presumptive PTB was also taken in CHC—Tamnar.

### Ethical declaration

The study protocol was approved by the Institutional Scientific Advisory Committee and the Institutional Ethics Committee, Indian Council of Medical Research National Institute of Research in Tribal Health, Jabalpur, Madhya Pradesh, India. Informed written consent was obtained from all the subjects who participated in the study. Patients with acute medical conditions/illnesses were treated on the spot, while other patients requiring prolonged treatment were referred to the nearest government health center. All procedures followed were in accordance with the ethical standards of the responsible committee on human experimentation (institutional and national) and with the Helsinki Declaration of 1975, as revised in 2000. This article does not contain any studies with animals performed by any of the authors.

### Statistical analysis

All the data were entered into Microsoft Excel and analyzed on Statistical Software for Social Sciences (IBM SPSS 25.0, IBM SPSS Statistics for Windows, version 25.0, Armonk, NY: IBM Corp). Data are presented as numbers and percentages for the discrete variables. Chi-square or Fisher's exact test was used to assess the significant difference in frequency between the groups. Univariate and multivariate analyses were performed to identify the independent risk factor for communicable and non-communicable diseases. A *P*-value of < 0.05 was considered significant. Data analysis was done by RK.

## Results

Most of the families were nuclear families (56.1%), with an average family size of 5.3. The majority (59.0%) of houses were kutcha houses, followed by semi-pucca 33.4%, and only 7.6% of houses were pucca houses (Table 1). Of the 909 families, 448 (49.3%) were tribal families. The overall prevalence of those regularly consuming alcohol was 6.2% while 6.5% were or had been smokers. The majority of smokers were men. Among the smokers, 88.1% were current smokers while 11.9% were ex-smokers. More than 90% of people had toilet facilities in their homes while 6.9% of people were still practicing open-field defecation (OFD).

**Table 1:** Distribution of socio-demographic factors, nutritional status, tobacco use, alcohol consumption, and dietary pattern of adults, Tamnar block (*n* = 909).

**Parameter**	**Number (%)**
**Socio-demographic factors**	
**Age (in years)**	
15–24 years	119 (13.09)
25–44 years	634 (69.7)
45–64 years	128 (14.08)
65 and above	28 (3.08)
**Gender**	
Male	230 (25.3)
Female	679 (74.7)
**Type of house** ^*^	
Kutcha	536 (59.0)
Semi-pucca	304 (33.4)
Pucca	69 (7.6)
**Category**	
Tribe	448 (49.3)
Non-tribe	461 (50.7)
**Nutritional status**	
**Body mass index**	
≤ 18.5 Kg/*m*^2^	280 (31.0)
18.5–22.9 Kg/*m*^2^	385 (42.6)
23.0–24.9 Kg/*m*^2^	108 (11.9)
≥25.0 Kg/*m*^2^	131 (14.5)
**Tobacco use and alcohol consumption**	
Tobacco use	59 (6.5)
Alcohol consumption	56 (6.2)
**Dietary pattern**	
Vegetarian	666 (73.3)
Non-vegetarian	243 (26.7)

* For the categorization of housing into kutcha, semi-pucca, or pucca, the following are considered: type of roof—concrete, metal sheet, straw/bamboo; type of wall—concrete, wooden, bamboo/mud; and type of floor—concrete, wooden, clay, respectively.

Of the 909 individuals, 280 (31.0%) had a BMI of < 18.5 Kg/m^2^, suggesting the presence of chronic energy deficiency. It is interesting to note that more than 25% of individuals were either overweight or obese in the study cohort (Table 1).

Acute dermatitis and acute respiratory infections (ARIs) were common morbidities observed in the study cohort. Men were at higher risk of both acute dermatitis [*p* = 0.036, OR = 2.132, 95% confidence interval (CI) = 1.106–4.111] and acute respiratory infection (*p* = 0.013, OR = 2.562, 95% CI = 1.269–5.171) than women (Table 2).

**Table 2:** Distribution of the presence of different morbidities among adults of Tamnar block (*n* = 909).

**Disease**	**Total (909)**	**Male (230)**	**Female (679)**	***P*, OR (95%CI)**	**Tribe (448)**	**Non-tribe (461)**	***P*, OR (95%CI)**
ARI	33 (3.6)	15 (6.5)	18 (2.7)	0.01, 2.56 (1.26–5.17)	15 (3.3)	18 (3.9)	0.72, 0.85 (0.42–1.71)
Diarrhea/dysentery	8 (0.9)	2 (0.9)	6 (0.9)	1.00, 0.98 (0.19–4.90)	2 (0.4)	6 (1.3)	0.28, 0.34 (0.06–1.69)
Scabies	11 (1.2)	3 (1.3)	8 (1.2)	1.00, 1.10 (0.29–4.21)	5 (1.10)	6 (1.3)	1.00, 0.85 (0.25–2.82)
Fungal infection	39 (4.3)	16 (7.0)	23 (3.4)	0.03, 2.13 (1.10–4.11)	20 (4.5)	19 (4.1)	0.87, 1.08 (0.57–2.06)
Hypertension^#^	194 (21.7)	77 (34.2)	117 (17.5)	< 0.00, 2.44 (1.74–3.34)	94 (21.4)	100 (22.1)	0.87, 0.96 (0.70–1.32)
T2DM	36 (4.0)	18 (7.8)	18 (2.7)	0.00, 3.11 (1.59–6.10)	12 (2.7)	24 (5.2)	0.06, 0.50 (0.24–1.01)
Cerebro-cardiovascular events	11 (1.2)	9 (3.9)	2 (0.3)	< 0.01, 13.78 (2.95–64.28)	4 (0.9)	5 (1.1)	1.00, 0.82 (0.21–3.08)
TB	23 (2.5)	11 (4.8)	12 (1.8)	0.02, 2.79 (1.21–6.41)	13 (2.9)	10 (2.2)	0.53, 1.34 (0.58–3.10)
Leprosy	11 (1.2)	5 (2.2)	6 (0.9)	0.15, 2.49 (0.75–8.24)	6 (1.3)	5 (1.1)	0.77, 1.23 (0.37–4.08)
Fluorosis	11 (1.2)	07 (3.0)	04 (0.6)	0.00, 5.29 (1.53–18.26)	6 (1.3)	5 (1.1)	0.77, 1.23 (0.37–4.08)
Other diseases	77 (8.5)	21 (9.1)	56 (8.2)	0.68, 1.11 (0.66–1.89)	28 (6.3)	49 (10.6)	0.02, 0.56 (0.34–0.90)

# Blood pressure was monitored in 892 people (225 men, 667 women) only.

During the survey period of 1 year, a total of 23 individuals were suffering from pulmonary tuberculosis (four participants having already completed treatment, 13 were on anti-tubercular treatment, and six were identified through sputum examination during the survey). Patients with a confirmed tuberculosis diagnosis were followed up and referred to the nearest Directly Observed Treatment- Short Course (DOTS) center for further management. Men had a significantly higher prevalence of tuberculosis than women (4.8 vs. 1.8%, *p* = 0.025, OR = 2.792, 95% CI = 1.215–6.417) (Table 2). Multibacillary (MB) leprosy and scabies were also observed in 11 (1.2%) study participants. No malaria parasite was identified in 303 individuals having fever during the survey period (including monsoon and post-monsoon seasons from June to November 2019). No significant difference in the prevalence of any communicable disease was observed between tribe and non-tribe communities (Table 5).

Of the study participants, high blood pressure and type II diabetes were present in 21.8 and 4% of the study subjects, respectively. Men had statistically significantly higher risk for both hypertension (*p* < 0.0001, OR = 2.446, 95% CI = 1.741–3.346) and type II diabetes (*p* = 0.001, OR = 3.118, 95% CI = 1.593–6.102). Furthermore, men had an approximately five-times higher prevalence of fluorosis than women (3.0 vs. 0.6%, *p* = 0.008, OR = 5.297, 95% CI = 1.536–18.264). There was no significant difference in the prevalence of hypertension, type II diabetes, and fluorosis between tribe (*n* = 448) and non-tribe (*n* = 461) communities. Eleven (1.6%) subjects had a history of cerebro-cardiovascular events in their lifetime, of which nine were male (Table 2).

Emaciation was seen in 39 (4.2%) individuals. The presence of clinically observed anemia (pallor) was suspected in 253 (27.8%) individuals. Of these 253 anemic cases, 241 (95.3%) were female. A complete blood count with a peripheral smear to confirm the diagnosis and type of anemia could not be done. Other signs of nutritional deficiency such as sparse hair (*n* = 6), moon face (*n* = 3), knock knee (*n* = 3), bow leg (*n* = 3), and marasmus (*n* = 1) were seen. Goiter was also seen in two individuals. We were not able to assess the protein, vitamin, and mineral levels associated with these nutritional deficiency signs.

Applying multivariate analysis, it was found that being male, smoking, and having nutritional deficiencies were independent risk factors for communicable diseases (Table 3). Similarly, being male, having a high BMI (>25 Kg/m^2^), disturbed sleep, smoking, and nutritional deficiencies were independent risk factors for the occurrence of any non-communicable disease (Table 4). Whether belonging to a tribal community or not, the type of house a person lives in (kutcha/pucca/semi-pucca), living in a joint or nuclear family, alcohol use, and having a vegetarian or non-vegetarian diet did not have a significant impact on the occurrence of communicable or non-communicable diseases (Tables 3, 4).

**Table 3:** Risk factors associated with the communicable disease among study participants.

**Category**	**Total (%)**	**Univariate analysis**	**Multivariate analysis**
		***p*** **, OR (95%CI)**	***p*** **, OR (95%CI)**
**Sex**			
Male	51 (22.2)	< 0.01, 2.13 (1.44–3.14)	0.01, 1.96 (1.25–3.06)
Female	80 (11.8)	–	–
**BMI**			
< 18.5 Kg/m^2^	56 (20)	0.01, 1.79 (1.17–2.74)	0.07, 1.51 (0.96–2.37)
18.5–22.9 Kg/m^2^	47 (12.2)	–	–
23–24.9 Kg/m^2^	15 (13.9)	0.62, 1.16 (062–2.16)	0.59, 1.19 (0.62–2.27)
≥25 Kg/m^2^	13 (9.9)	0.53, 0.79 (0.41–1.51)	0.42, 0.76 (0.38–1.49)
**Ethnicity**			
Tribe	63 (14.1)	0.77, 0.94 (0.61–1.37)	0.71, 0.93 (0.62–1.37)
Non-tribe	68 (14.8)	–	–
**Type of house**			
Kutcha	82 (15.3)	0.72, 1.20 (0.57–2.52)	0.51, 1.30 (0.59–2.86)
Semi-pucca	40 (13.2)	1.00, 1.01 (0.46–2.19)	0.66, 1.20 (0.53–2.71)
Pucca	09 (13)	–	–
**Type of family**			
Nuclear	76 (14.9)	0.70, 1.09 (0.75–1.59)	0.66, 1.09 (0.73–1.61)
Joint	55 (34.4)	–	–
**Diet**			
Non-vegetarian	94 (14.1)	0.67, 0.91 (0.60–1.38)	0.57, 0.88 (0.57–1.36)
Vegetarian	37 (15.2)	–	–
**Sleep**			
Disturbed	18 (19.1)	0.16, 0.68 (0.39–1.17)	0.40, 1.28 (0.71–2.33)
Regular	113 (13.9)	–	–
**Smoking**			
Yes	17 (28.8)	0.01, 2.61 (1.43–4.74)	< 0.01, 3.94 (2.16–7.16)
No	114 (13.4)	–	–
**Alcohol**			
Yes	12 (21.4)	0.16, 1.68 (0.86–3.27)	0.98, 0.99 (0.44–2.19)
No	119 (14)	–	–
**Nutrient deficiency**			
Yes	24 (39.3)	< 0.01, 4.49 (2.58–7.80)	< 0.01, 3.94 (2.16–7.16)
No	107 (12.6)	–	–

**Table 4:** Risk factors associated with the non-communicable disease among study participants.

**Category**	**Total (%)**	**Univariate analysis**	**Multivariate analysis**
		***p*** **, OR (95%CI)**	***p*** **, OR (95%CI)**
**Sex**			
Male	98 (42.6)	< 0.01, 2.73 (1.98–3.76)	< 0.01, 2.37 (1.62–3.45)
Female	145 (21.4)	–	–
**BMI**			
< 18.5 Kg/m^2^	46 (16.4)	0.01, 0.60 (0.40–888)	0.01, 0.48 (0.31–0.75)
18.5–22.9 Kg/m^2^	95 (24.7)	–	–
23–24.9 Kg/m^2^	35 (32.4)	0.10, 1.46 (0.91–2.33)	0.10, 1.51 (0.923–2.470)
≥25 Kg/m^2^	62 (47.3)	< 0.01, 2.74 (1.81–4.15)	< 0.01, 2.66 (1.70–4.15)
**Ethnicity**			
Tribe	113 (25.2)	0.33, 0.85 (0.64–1.15)	0.43, 0.87 (0.62–1.22)
Non-tribe	130 (28.2)	–	–
**Type of house**			
Kutcha	140 (26.1)	0.04,0.58 (0.34–0.98)	0.59, 0.85 (0.48–1.52)
Semi-pucca	77 (25.3)	0.051, 0.56 (0.32–0.97)	0.25, 0.70 (0.39–1.28)
Pucca	26 (37.7)	–	–
**Type of family**			
Nuclear	140 (27.5)	0.59, 1.08 (080–1.46)	0.75, 1.05 (0.75–1.46)
Joint	103 (25.8)	–	–
**Diet**			
Non-veg	176 (26.4)	0.73, 0.94 (0.67–1.31)	0.69, 0.93 (0.64–1.33)
Veg	67 (27.6)	–	–
**Sleep**			
Disturbed	50 (53.2)	< 0.01, 3.66 (2.36–5.64)	< 0.01, 3.35 (2.09–5.38)
Regular	193 (23.7)	–	–
**Smoking**			
Yes	33 (55.9)	< 0.01, 3.86 (2.26–6.61)	0.01, 2.23 (1.14–4.342)
No	210 (24.7)	–	–
**Alcohol**			
Yes	25 (46.6)	0.01, 2.34 (1.35–4.06)	0.93, 1.02 (0.51–2.05)
No	218 (25.6)	–	–
**Nutrient deficiency**			
Yes	27 (44.3)	0.01, 2.32 (1.370–3.941)	0.00, 2.72 (1.45–5.09)
No	216 (25.5)	–	–

**Table 5:** Percent prevalence of diseases in the tribe and non-tribe study population.

**Disease**	**Tribe (448)**	**Percentage**	**Non-tribe (461)**	**Percentage**	**Total (909)**	**Percentage**	***P*-value**
BMI (< 18.5kg/m^2^)	141	31.4%	139	30.1%	280	30.8%	0.66
ODF	35	7.8%	28	6.0%	63	6.9%	0.36
Anemia	111	24.7%	129	27.9%	240	26.4%	0.74
ARI	97 (11.2)	21.6%	77 (9.1)	16.7%	174 (10.2)	19.1%	0.15
Diarrhea/dysentery	08 (0.9)	1.7%	17 (1.6)	3.6%	22 (1.3)	2.4%	0.20
Scabies	09 (1.0)	2%	10 (1.2)	2.1%	19 (1.1)	2.0%	0.82
Fungal infection	30 (3.5)	6.69%	24 (2.87)	5.2%	54 (3.2)	5.9%	0.49
Hypertension	94	20.98%	100	21.6%	194	21%	0.87
T2DM	11 (2.4)	2.45%	24 (5.1)	5.2%	35 (3.8)	3.8%	0.03
Cardiovascular events	4 (0.9)	0.89%	5 (1.1)	1.0%	9 (1.0)	0.9%	1.00
TB	14 (3.1)	3.1%	10 (2.2)	2.1%	24 (2.6)	2.6%	0.41
Leprosy	07 (0.5)	1.5%	06 (0.7)	1.3%	13 (0.8)	1.4%	1.0
Worm infection	12 (1.4)	2.67%	10 (1.2)	2.1%	22 (1.3)	2.4%	0.8
Fluorosis	19 (2.2)	4.24%	10 (1.2)	2.1%	29 (1.7)	3.1%	0.13
Mental illness	03 (0.3)	0.66%	11 (1.3)	2.3%	14 (0.8)	1.5%	0.03
Other diseases	69 (8.0)	15.4%	96 (11.3)	20.8%	165 (9.6)	18.1%	0.02

## Discussion

In this study, community-wide disease profiling was done among residents of industrial (mining) areas of Tamnar block, Raigarh, Chhattisgarh, India, and various types of communicable diseases, such as acute respiratory illness, pulmonary tuberculosis, leprosy, and scabies, were observed. Non-communicable diseases, such as hypertension and type II diabetes, were also observed. The National Health Family Survey 5 (NFHS-5) reported the prevalence of hypertension in men (24%) and women (21%). The frequency of communicable and non-communicable diseases was similar in tribe and non-tribe communities. Previous recent studies conducted with different tribe communities also reported the same (4–7). Because they are chronic in nature, non-communicable diseases put a continuous extra economic burden on marginalized tribal communities and also on the nation.

The NFHS-5 in India reported a 25.3 and 19.4% prevalence of being underweight among women and men, respectively, residing in the rural parts of Chhattisgarh, which are similar findings to our study. However, these results are considerably lower than the *National Nutrition Monitoring Bureau* (NNMB) 2009 tribal survey of Madhya Pradesh and Chhattisgarh state (8). This improvement could be due to an improved public distribution system. As the public distribution system (PDS) evolved as a system of management of scarcity through the distribution of food grains at affordable prices, PDS is operated under the joint responsibility of the central and the state/UT governments. The central government, through the Food Corporation of India (FCI), has assumed the responsibility for the procurement, storage, transportation, and bulk allocation of food grains to the state governments.

During the study period and 2 weeks before the survey, a high prevalence of acute respiratory infection (3.6%) was observed. This was higher than that reported by the NFHS-5 Chhattisgarh report, where it was 1.5%. Previous studies also reported a high prevalence of ARI in coal mine workers due to exposure to high levels of particulate matter (9). In-depth studies need to be conducted for delineating possible other causes of infections. In this study, the prevalence of tuberculosis is estimated to be 564/100,000 of the population, which is above the national rate (2018) of 199/100,000 and that of Chhattisgarh 295/100,000 (2018) (8). High tuberculosis prevalence has also been reported in various tribal groups from previous studies (10–12). We did not find any difference in tuberculosis prevalence between tribe and non-tribe communities. Furthermore, long-term inhalation of coal mine dust is responsible for several types of respiratory diseases such as classic coal workers' pneumoconiosis (CWP) and silicosis (13), which are predisposing risk factors for tuberculosis (2, 14).

Multibacillary leprosy was also observed in study participants (1.2%). Previous studies have also shown the high burden of leprosy in tribes. Measures for timely detection and further management of new leprosy cases in tribal areas need to be adopted (15–17). Acute contact dermatitis was seen in 4.0% of the study participants, with a high prevalence in men. Various causes such as occupational hazards, environment, overcrowding, and poor living conditions may be major factors (3). Furthermore, there are seminal pieces of evidence showing that coal mine workers are at a higher risk of dermatitis (18). A report by the Council of Scientific and Industrial Research-National Environmental Engineering Research Institute (2018) reports the presence of alarmingly high levels of arsenic in drinking water in 14 villages of Tamnar block (19), and the high arsenic content in water is associated with the development of dermatitis with superimposed fungal infection (20, 21).

A high prevalence of hypertension was observed in both men and women in the present study, which is contrary to the NFHS-5 survey, which reported a prevalence of hypertension in men (24%) and women (21%) (22). The cumulative prevalence of hypertension in the tribe communities interviewed in the present study was 21.4%. Rizwan et al. (23) performed a meta-analysis and estimated a 22.5% prevalence of hypertension in Indian tribe communities. Similarly, a high prevalence of hypertension among tribe communities was also reported by Chakma et al. (7) and Indian Council of Medical Research-National Institute of Nutrition (24) and the *National nutrition monitoring bureau* (NNMB). On account of the high prevalence of hypertension in different communities of India, to reduce the prevalence of hypertension by 25%, the Ministry of Health, Government of India, together with WHO-India, state governments, and Resolve to Save Lives initiated a nationwide “Indian hypertension control initiative” program in November 2017 (25). The program has been started in 25 districts across India. The NFHS-5 reported a 12% and 14% prevalence of diabetes among men and women, respectively, across India (22), which is much lower than the prevalence in the present study, where the prevalence of type II diabetes in women and men is 2.7 and 7.8%, respectively. Upadhyay et al. (26), after doing a meta-analysis, reported a 5.9% prevalence of type II diabetes in tribe communities. The higher prevalence of type II diabetes in study participants is a matter of concern, as studies have shown that patients with diabetes and a low socioeconomic status are more prone to diabetes-associated morbidities, as they have poor access to quality healthcare services (27). Previous studies have also shown that individuals performing mining activities are at a higher risk for obesity, diabetes, and hypertension than the general population (28), and therefore, there is a need to strengthen the activities of the National Programme for Prevention and Control of Cancer, Diabetes, Cardiovascular Diseases and Stroke (NPCDCS), particularly in villages adjacent to mining areas (29).

In this study, men have a higher prevalence of most communicable and non-communicable diseases than women. Previous studies have also shown that men are at a higher risk for non-communicable diseases. The reason for the high prevalence of diseases such as ARI, contact dermatitis, and tuberculosis in men is their tobacco smoking behavior and exposure to high aerosols/particulate matters while working in mines (30, 31).

It is interesting to note that as the BMI of individuals increases, the risk for non-communicable disease also increases while the risk of communicable diseases decreases (Tables 3, 4). This is in concordance with previous reports (32–35). Therefore, it is essential to maintain a BMI in the normal range by having a healthy diet and taking regular physical exercise. Undernutrition was also found as one of the independent risk factors for communicable and non-communicable diseases, which agrees with previous reports (36). Several national-level policies and programs are run by the Government of India from time to time to tackle the problem of undernutrition. Recently, the National Nutrition Mission (*POSHAN Abhiyaan*) (37) was launched to reduce child and maternal malnutrition. There is also a need for national-level programs to reduce undernutrition in adult men and women, and to reduce the burden of communicable and chronic non-communicable diseases.

Irregular sleep patterns were identified as an independent risk factor for non-communicable diseases, which is in concordance with previous studies. In a recent longitudinal study with rural-dwelling South African people, shorter and poor-quality sleep were found to be associated with hypertension (38). The plausible cause for irregular sleep in the study cohort may be the changing working-duty hours (shift duty) of either the study participants or their family members and also the large movement of heavy-duty vehicles for transportation of coals from mines to plants.

There are certain limitations to this study. First, there was an unequal amount of men and women who participated in the study. The main reason for this was that most men were not present at the house during the interview. Second, spirometry could not be done in individuals with symptomatic chronic obstructive pulmonary disease (COPD). The distance between Tamnar block and the district hospital and the actual time to reach the district hospital were the main reasons for non-compliance with spirometry. The status of anemia could not be checked. Further, nutritional deficiencies were only considered based on clinical signs and symptoms. The assays for nutrient deficiencies such as iron, calcium, and vitamin D could not be performed.

In conclusion, comprehensive disease profiling is needed on a large scale at different time intervals to generate data on disease prevalence, which will help policymakers to adopt control, prevention, and management measures regarding the health problems of individuals exposed to different health hazards. In this study, nutritional deficiency is identified as an independent risk factor for both communicable and non-communicable diseases. National-level programs should be strengthened to improve the nutritional status and reduce the burden of chronic diseases. Community participation approach in such remote places is may be an effective way to extend healthcare system.

## Data availability statement

The original contributions presented in the study are included in the article/Supplementary material, further inquiries can be directed to the corresponding author.

## Ethics statement

The studies involving human participants were reviewed and approved by Institutional Ethical Committee, ICMR-National Institute for Research in Tribal Health. The patients/participants provided their written informed consent to participate in this study.

## Author contributions

SS, RS, and TC designed the study. SS, SK, AG, and AK have done field work and collected the demographic and clinical data. SS and RK did the literature search and drafted the manuscript. RS, RK, and AK were involved in statistical work. TC, HK, and MD critically reviewed the manuscript for important intellectual content. All authors approved the final version of the manuscript.
