# Supplementary material for: Health profile of people living in the Gare Palma mining area of Tamnar block, Raigarh, Chhattisgarh, India
Source: Front Public Health. 2023 Mar 21;11:1010025. doi: 10.3389/fpubh.2023.1010025 (PMC10072258; doi:10.3389/fpubh.2023.1010025)
Supplement: Supplementary file 1 [file Data_Sheet_1.docx]

**Operational Definitions used in study**

**Cerebrocardiovascular disease (CVD):** Conditions such as ischemic stroke or mini-stroke and sometimes a haemorrhagic stroke or history of ischemic heart disease (IHD)/acute myocardial infarction (AMI)/ Angina Pectoris were classified as Cerebrocardiovascular disease (CVD).

**Hypertension**: Systolic and diastolic blood pressures were measured in sitting posture using a fully automatic digital Blood Pressure Monitor (Omron HEM 7120). Individuals with systolic blood pressure of >140 mm Hg and diastolic pressure of >90 mm Hg was considered as hypertensive or taking antihypertensive drugs prescribed by registered medical practitioner.

**Chronic Obstructive Pulmonary Diseases** (COPD) was defined as chronic expiratory airflow limitation that is not fully reversible. It was considered if patients are having any two sign and symptoms like (i) shortness of breath (especially during physical activities), (ii) wheezing, (iii) chest tightness, (iv) a chronic cough that may produce mucus or sputum, for more than three months and negative for tuberculosis, (v) frequent respiratory infection, (vi) positive signs in spirometry (done in district hospitals and examined by chest physician) and (vii) getting relief from beta blockers or other bronchodilators.

**Diabetes** was considered if there is symptoms of diabetes and random plasma glucose ≥ 200 mg/dl and confirmation with another test subsequently and any patient taking oral hypoglycaemic drugs or Insulin after consultation with registered medical practitioner. ^7^

**Dental and Skeletal Fluorosis**: For the identification of dental fluorosis ICMR index were used for dental fluorosis. ^8^ Skeletal fluorosis was defined if any positive signs elicited by four physical exerciseplus presence of increased levels of fluoride in drinking water and urine.^9^

**Acute contact dermatitis:** Any sign of acute inflammation of the skin such as pruritus, erythema, blistering and ulceration.

**Smoker**: A person who smoked/ used tobacco in any form either daily or occasionally in the previous 30 days.

**Alcohol abuser:** A person who consume alcohol in any form either daily or occasionally in previous 30 days.

**Sleep Disturbance:** Individual complaining of "trouble sleeping," (when the patient having difficulty in falling asleep) or once asleep, he/she were not able to remain asleep for the desired length of time (for minimum 6 hrs) were classified as individual having disturbed sleep.

**BMI group classification:** Individuals having BMI> 25 Kg/m^2^ were categorised as obese, between 23-25 Kg/m^2^ overweight and <18.5 Kg/m^2^ were having chronic energy deficiency (CED) as per consensus statements for diagnosis obesity in Asian Indian subjects.
